# Optimization and parallelization of the discrete ordinate method for radiation transport simulation in OpenFOAM: Hierarchical combination of shared and distributed memory approaches

**DOI:** 10.12688/openreseurope.13017.1

**Published:** 2021-03-24

**Authors:** Jose Moreno-SanSegundo, Cintia Casado, David Concha, Antonio S. Montemayor, Javier Marugán

**Affiliations:** 1 Department of Chemical and Environmental Technology, Universidad Rey Juan Carlos, Móstoles, Madrid, 28933, Spain; 2 Department of Computer Science and Statistics, Universidad Rey Juan Carlos, Móstoles, Madrid, 28933, Spain

**Keywords:** Photochemical reactors, CFD, multithreaded computing, shared memory, distributed memory, hierarchical parallelization

## Abstract

This paper describes the reduction in memory and computational time for the simulation of complex radiation transport problems with the discrete ordinate method (DOM) model in the open-source computational fluid dynamics platform OpenFOAM. Finite volume models require storage of vector variables in each spatial cell; DOM introduces two additional discretizations, in direction and wavelength, making memory a limiting factor. Using specific classes for radiation sources data, changing the store of fluxes and other minor changes allowed a reduction of 75% in memory requirements. Besides, a hierarchical parallelization was developed, where each node of the standard parallelization uses several computing threads, allowing higher speed and scalability of the problem. This architecture, combined with optimization of some parts of the code, allowed a global speedup of x15. This relevant reduction in time and memory of radiation transport opens a new horizon of applications previously unaffordable.

## Plain language summary

A significant improvement in the computational time required for the simulation and design of photochemical processes is reported. Notable upgrades have been introduced on the previous computational codes available in the open-source platform OpenFOAM. This is a software for the rigorous resolution of complex mathematical problems involving fluid flow, heat flow, chemical reactions and radiation transport phenomena, among others. In particular, the reported work is focused on the enhancement of the Discrete Ordinate Method (DOM), which is an algorithm for solving radiation transport systems (such as those involved in photochemical processes with solar light, LED, etc.). A global speedup of x15 has been achieved. This relevant reduction in time and memory of radiation transport opens a new horizon of applications previously unaffordable.

## Symbol list

*e ^a^*          Absorbed energy (W m ^-3^)

Ψ          Unitary dimensionless time

$I_{\lambda ,\vec{\text{Ω}}}$ Radiation intensity at wavelength *λ*, and direction $\vec{\text{Ω}}$ (W m ^-2^)

*κ _λ_*        Absorption coefficient at wavelength *λ* (m ^-1^)

*σ _λ_*        Scattering coefficient at wavelength *λ* (m ^-1^)

*σ*          Steffan-Boltzmann constant, 5.67 ×10 ^-8^ W m ^-2^ K ^-4^

$p(\vec{{\text{Ω}}^{\prime }}\;\to \;\vec{\text{Ω}})$ Scattering phase function between directions $\vec{\text{Ω}}$ and $\vec{{\text{Ω}}^{\prime }}\;$

## Introduction

The rigorous simulation of radiation transport in participating media requires a huge computational time, as it involves discretization not only in time and space as most physical phenomena, but also in direction and wavelength. Therefore, simplification is usually required to encompass the challenge. Most of the available models avoid discretization in wavelength or use a coarse discretization in a few discrete intervals, with no coupling between them. Most of the technical applications of radiation transport simulation can reach the expected precision with the simplest models that avoid directional discretization in exchange for a lower precision. Another possibility is discarding some of the more computational costly optical phenomena. The radiosity model, widely extended in graphical illumination, assumes transparent media and full diffuse radiation, while in P-N models, used in thermal applications, surfaces do not reflect nor transmit radiation. In contrast, photoactivated processes, such as photochemical reactions triggered by the absorption of light by some reactants or catalytic species, usually require the simulation of optical phenomena such as anisotropic scattering, specular reflection, and anisotropic light sources. In these cases, the use of simplified models is hindered, and more rigorous approaches, such as the discrete ordinate method (DOM) or the photon Monte Carlo (PMC) are required to cover all the optical phenomena involved in the correct evaluation of the radiation field of photoactivated systems.

Serial simulation in a single thread of radiation transport in photochemical systems with scattering media using a high precision setup for the resolution of meshes above 200k cells with an angular discretization of at least 10×10 could require up to 24 hours of computational time in a single processor to reach convergence in steady-state, or per time-step in a transient simulation. Therefore, parallelization of the computation is critical to reduce time consumption. PMC is a finite element method (FEM), easy to parallelize, as every photon resolution is independent of others. In contrast, DOM is a finite volume method (FVM), with higher serial performance, and easier to couple with other models, but harder to parallelize due to the strong interaction between simulation data.

Most of the FVM simulation frameworks widely used such as ANSYS Fluent, Comsol and OpenFOAM ^1^ use a distributed memory (DM) parallelization strategy. It splits the mesh into several regions, each computing node solves a region, and sends the values of the fields in the interfaces to neighbour nodes. In DM approaches, the processing cores can reside in different machines, but the communication time between nodes is added to the overall computation time. This communication time grows with the number of processes, while the resolution time decreases, reaching a critical number, where a new process increases the total time instead of reducing it. This limit depends on the specific simulation and would grow with the number of cells.

Some DM parallelization schemes, such as OpenMPI, MPICH, Intel MPI or Microsoft MPI, evolved to adapt to a paradigm of multi-core processors using the shared main memory to improve performance ^2^, but traditional shared memory (SM) approaches also allow the use of the processor cache, which is dozens of times faster.

Whereas SM parallelization alone has limited scalability, as it is restricted to a single machine, a hierarchical combination of both approaches where every DM process is composed of several SM threads is proposed to overcome this computational limitation. The parallel-computation enhanced DOM model has been profiled to study memory and time consumption of each step in the algorithmic implementation in OpenFOAM before and after each enhancement. Validation of the performance improvement has been done through the total computational time required for the simulation of three different photochemical processes, using the standard DOM implementation in commercial software ANSYS Fluent as a benchmark.

The original DOM model implemented in OpenFOAM, fvDOM, was included in the first release of the simulation framework, developed by Henry Weller in the late 80’s. It was developed under a completely different computation paradigm, when access to memory was, in relative terms, compared to mathematical operations in the processor, hundreds of times faster than actuality. The model has remained unchanged since then.

The core of OpenFOAM is a masterwork piece using almost all C++ language features often and carefully, like templates, multiple heritage, macros and virtual features. It also hides the parallelization to new developers, so they can create a new model, written to be used serial, and use it parallel with no additional changes. In the authors’ opinion, this level of mastery and complexity in the core created an inertial resistance to change or update the pre-existing code and lead the efforts of the developing community to increase the platform possibilities (new models, interpolation schemes, auxiliary tools to mesh or postprocess results etc.), keeping the core unmodified.

In a recent report, we have presented the development of a novel DOM module ^3^ validated both in simple geometries and in real photochemical reactors with different types of light sources ^4^. This model, implemented on the OpenFOAM platform, improves the precision significantly in the simulation of solar and LED light sources, and it has been made open access and available to download ^5^. The present work aims to study the dramatic improvement achieved in the performance of the model using a novel computing parallelization scheme. There are no changes in the model precision, or scope, only in its performance. The optimized version is already available open access ^6^ under the GNU GPL v3 license.

To the best of our knowledge, previous reports in the literature on the optimization of the DOM in OpenFOAM are focused on changes in the algorithm ^7^ and not on optimizing the implementation to reduce time or memory. Only the work of Efremenko *et al.* ^8^ shows a first attempt to study and improve the parallelization of the DOM, but in a simple implementation independent of any simulation platform and limited to simple structured cubic meshes.

## Methods

The radiation model Discrete Ordinate Method for Radiative Transfer v 1.0 was developed as a new feature for OpenFOAM v7. Its optimized and parallelized version was labelled as v 2.0. Both code versions are available at the Github repository ^6^. Examples and case studies were run in an Intel Xeon E5-2630 v3 @ 2.40 GHz, 64 Gb RAM computer, under Ubuntu 14.02 Lts operative system.

Memory requirements of the models were analyzed under two different methods. The effect in total memory was measured using the Linux “free” command to get the available memory before starting and during the simulation. Memory requirements of the different classes were directly calculated considering the primitive types of the classes fields, and the memory required by pointers.

The time required by each method was measured using the OpenMP command “openmp_get_wtime()”. Profiled versions of both models were developed, with time flags after and before each measured method. However, these measurements and reports increase the computation time considerably. Thus, an unprofiled version of the code was used to evaluate global performance in the benchmark with ANYS Fluent.

A simple geometry with a growing number of cells was chosen in the profiling and methods evaluation, whereas a more accurate description complex geometry was used for the reactors selected to analyze the global performance. The meshes of all the studied cases are available at Zenodo repository ^9^.

ANSYS Fluent 2019 R2 DOM method was used as a benchmark in the global performance of the model. The simulations were run in the same machine, to discard hardware effects.

## Model description

### Discrete ordinate method (DOM)

**Figure 1.**
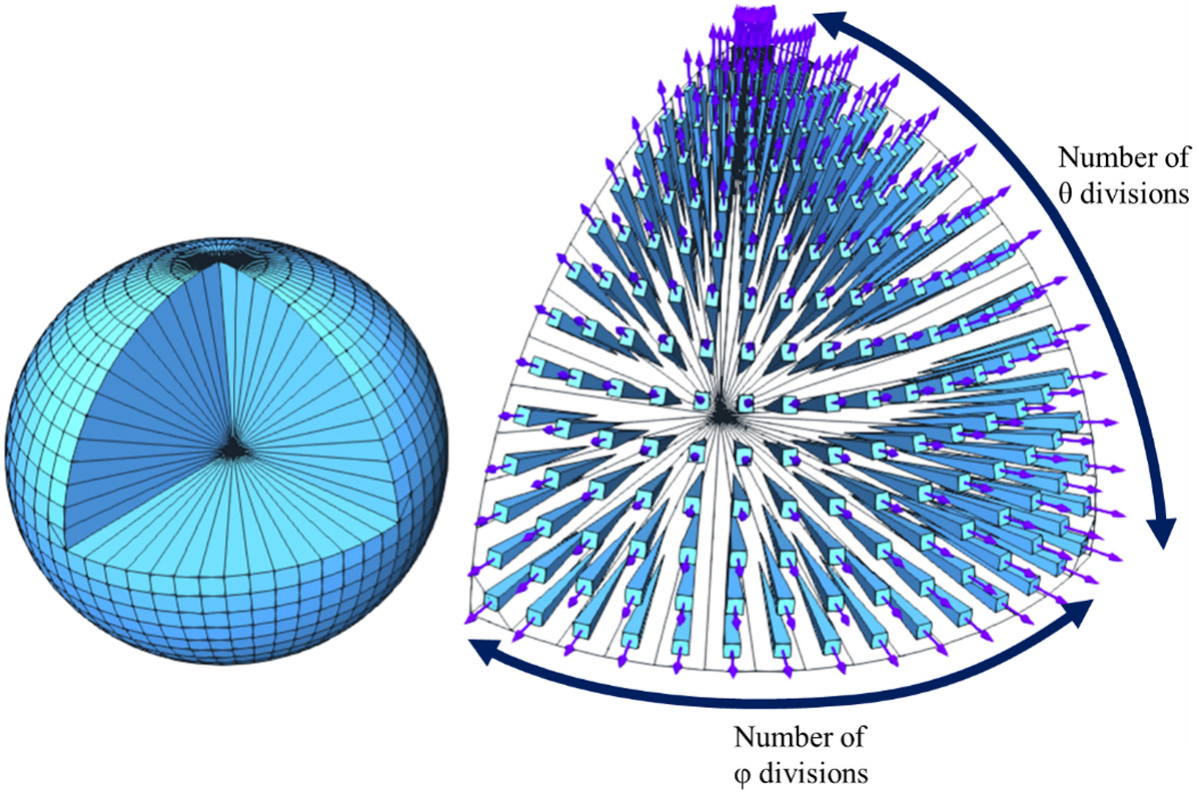
15×15 quadrature, detailing one of the octants.

The radiative transfer equation (RTE) ( Equation 1) describes the conservation of radiative intensity in a direction of space.


$$\;\frac{dI_{\lambda ,\vec{\text{Ω}}}}{ds}\;=\;\underset{Absorption}{\underset{︸}{-\kappa_{\lambda }I_{\lambda ,\vec{\text{Ω}}}}}\;\underset{\begin{array}{l} \;Out\;\\ Scattering \end{array}}{\underset{︸}{-\sigma_{\lambda }I_{\lambda ,\vec{\text{Ω}}}}}\;\underset{\begin{array}{l} \;Thermal\;\\ \;Emission \end{array}}{\underset{︸}{+\;\frac{\kappa_{\lambda }\sigma T^{4}}{\pi }}}\;\underset{In\;Scattering}{\underset{︸}{+\;\frac{\sigma_{\lambda }}{4\pi }\;\int_{{\text{Ω}}^{\prime }\;=4\pi }p(\vec{{\text{Ω}}^{\prime }\;}\to \;\vec{\text{Ω}})I_{\lambda ,\vec{\text{Ω}}}d\vec{{\text{Ω}}^{\prime }}\;I_{\lambda ,\vec{\text{Ω}}}}}\;(\text{Eq.}\;1)$$

where $I_{\lambda ,\vec{\text{Ω}}}$ is the intensity of photons with wavelength *λ*, travelling in the main direction of the solid angle $\vec{\text{Ω}}$ ; *κ _λ_* is the volumetric absorption coefficient; *σ _λ_* is the volumetric scattering coefficient; *σ* is the Stefan-Boltzmann constant; *T* is the temperature; and $p(\vec{{\text{Ω}}^{\prime }}\;\to \;\vec{\text{Ω}})$ is the phase function, which describes the distribution of scattered radiation. Solving the RTE involves solving all directions, which can be achieved by a previous integration through simplifications (Rosseland ^10^, model P-n ^11, 12^, view factors ^13^, method of moments ^14, 15^) or by a subsequent numerical integration, as does the DOM or PMC methods ^16, 17^. The DOM consists of separately solving a finite number of directions that are representative of all nearby addresses (discrete ordinates) distributed following a map, called quadrature (see Figure 1). Then, the integral is calculated as the sum of the values of the discrete ordinates in all the directions of the sphere.

### Implementation

**Figure 2.**
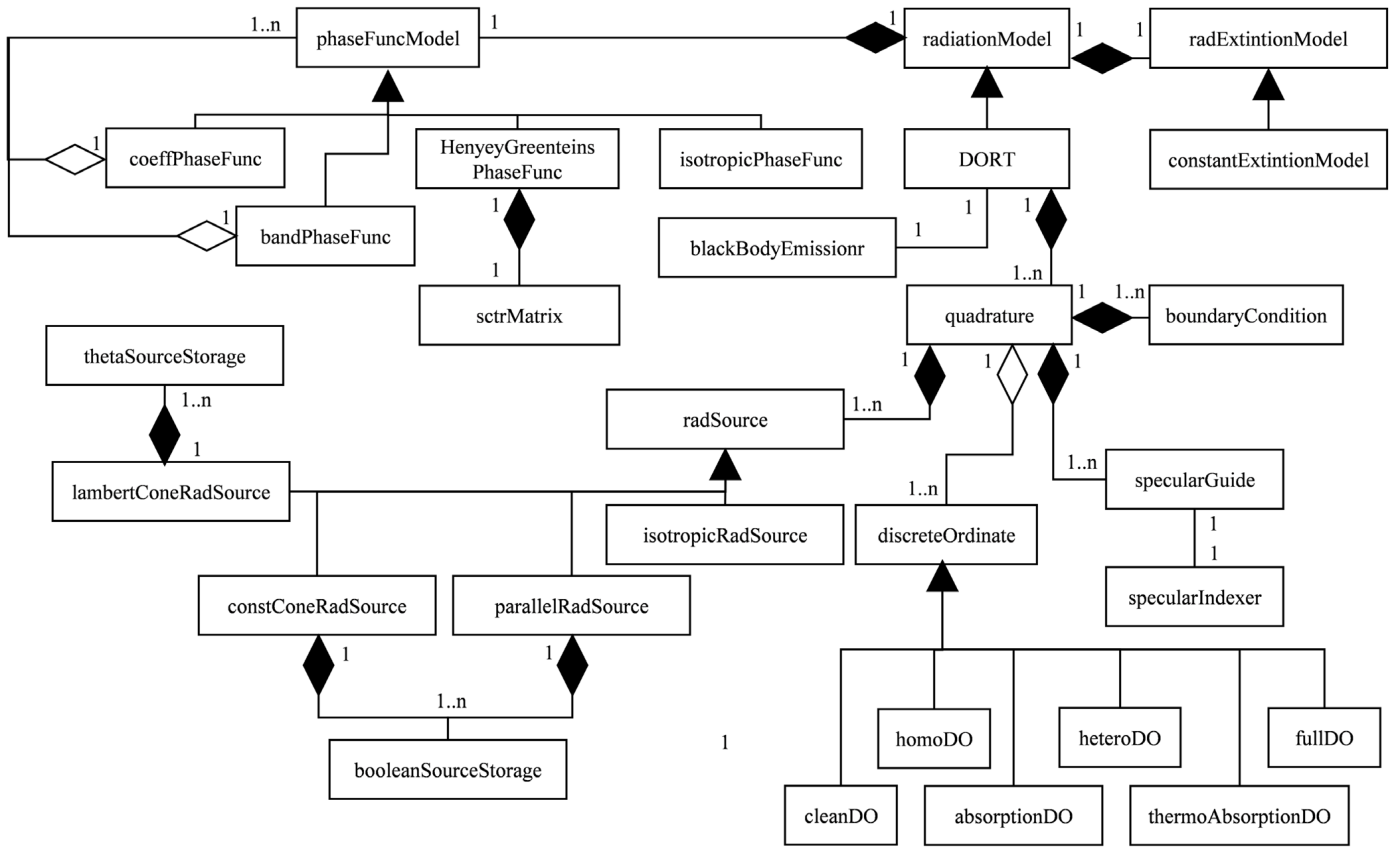
Unified Modeling Language class diagram of the DORT model.

The model, based on the original fvDOM, was developed in C++ under the classes structure of OpenFOAM represented in the Unified Modeling Language (UML) diagram shown in Figure 2. The main class, *DORT*, inherits from the OpenFOAM *radiationModel* class, and there is an instance per region in the simulation. Each region can have different optical properties, and *DORT* includes a reference to an *absorptionExtintionModel* class instance, that defines the distribution of both absorption and scattering coefficients and the chosen *phaseFunctionModel* describing the isotropic or anisotropic shape of the scattered radiation.

One of the enhancements included in the previously developed model ^3^ is the adaptation of the quadrature to the radiation sources, and there is the same amount of *quadrature* class instances than *radiationSource* ones. *DORT* class stores a list of references to pairs *quadrature*- *radiationSource*. *radiationSource* class stores the minimal information to calculate the intensity in each direction of the source, while *quadrature* not only defines the distribution map of the discrete ordinates, but also stores a list of pointers to all the *discreteOrdinate* class instances.

*absorptionExtintionModel*, *phaseFunctionModel* and *radiationSource* are abstract classes, using the predefined runTime tables in OpenFOAM to choose the proper inherited class during the initialization of the simulation. *discreteOrdinate* is also an abstract class, but the inherited class is chosen by quadrature, to minimize storage and calculation during the simulation (i.e. *cleanDiscreteOrdinate* is the simplest version, which only includes divergence in the RTE, avoids reading the optical properties, and lacks the in-scattering term). A more in-depth description of the model and its classes structure can be found elsewhere ^3^.

The peer-to-peer DM parallelization strategy was already implemented in OpenFOAM using the OpenMPI C++ API ^18^, while the SM approach uses the OpenMP C++ pragmas ^19^. SM parallelization uses the *fork-join* scheme, mainly parallelizing loops over cells or faces.

## Model profiling

**Figure 3.**
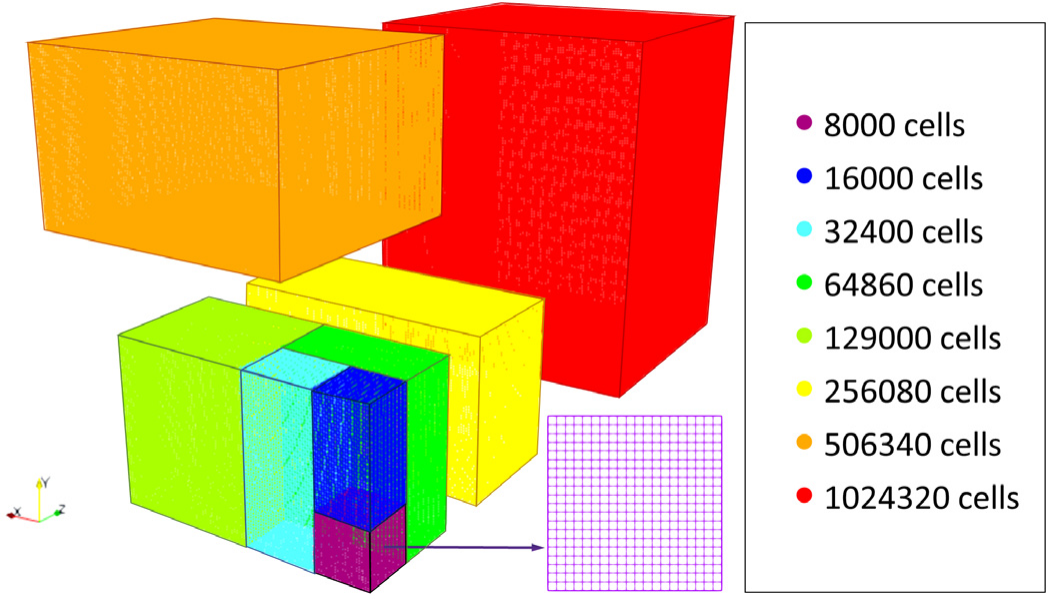
Profiling mesh regions.

The original model performance was profiled in meshes comprised of several regions with a range of cell numbers from 8000 to 1024000 ( Figure 3) to evaluate the operations where optimization can achieve a higher impact.

These eight meshes allowed study the computational time spent as a function of cell number in each of the 11 profiled program stages ( Table 1). Table 1 also shows the execution time of thesestages in an average 100 iterations serial simulation in 8 k and 506 k cells meshes. Stages 1 to 4 happen once at the beginning of the simulation, while the remaining stages are executed once per iteration.

**Table 1.** Model stages and contribution to simulation time in 8k and 506 k cells meshes.

	8000 cells mesh		506340 cells mesh	
	Time (s)	Contribution	Time (s)	Contribution
1. Mesh build	0.033	0.00%	2.063	0.00%
2. Build of instances	0.232	0.00%	3.188	0.00%
3. Build of the extinction model	0.215	0.00%	0.625	0.00%
4. Build of the scattering matrix	4.438	0.05%	4.438	0.00%
5. Global results cleaning	0.006	0.00%	0.430	0.00%
6. Global results generation	0.005	0.00%	0.560	0.00%
7. Quadrature results cleaning	0.002	0.00%	0.274	0.00%
8. Quadrature results generation	3.350	0.04%	155.000	0.04%
9. Operation between fluxes	2.106	0.02%	110.000	0.03%
10. In-scattering term update	8,699.998	95.91%	386,299.726	92.70%
11. Differential equation solving	360.350	3.97%	30,146.032	7.23%
Total	9,071 s (~2.5 hours)		416,722 s (4.8 days)	

**Figure 4.**
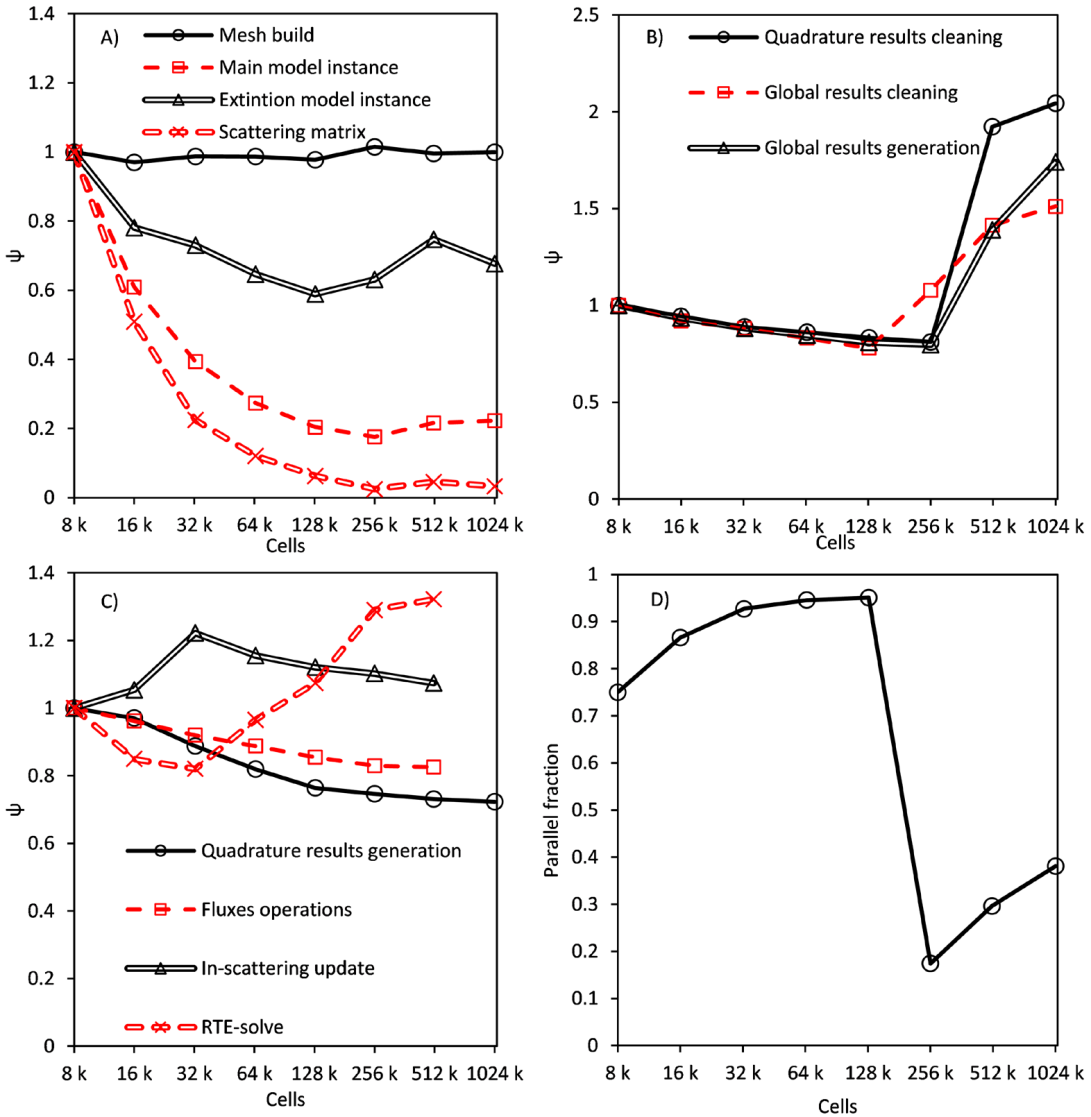
Time trend of stages: executed once per simulation ( **A**), once per iteration ( **B**), at least once per iteration and discrete ordinate ( **C**), parallel fraction of global results generation stage ( **D**).

Figure 4 shows the trends of the computational time before optimization of the code as a function of the number of cells in the mesh for the different algorithm stages. Direct results of time versus the number of cells are hard to analyze as every trend seems to be a line with a higher or lower slope. Therefore, a dimensionless unitary function of time has been defined to make easier the interpretation of the results using the mesh of 8000 cells as reference ( Equation 2).


$$\;\psi_{i}\;=\;\frac{t_{i}}{n_{cells_{i}}}\;\frac{8000}{t_{8000}}\;(\text{Eq.}\;2)$$

Figure 4A shows the results of stages 1 to 4, which happen just once at the beginning of the simulation. Their computational time is negligible in the global simulation time, but 98% of the total memory consumption of the model is allocated in these stages. Time trends can be easily explained. The mesh building stage needs a constant amount of time per cell in the geometry, scattering matrix needs a fixed amount of time no matters the size of the mesh (hyperbolic trend). Instancing stages are a combination of both.

Figure 4B displays the stages happening once per iteration (80–150 times in a simulation). The effect of these stages is slightly higher, but still irrelevant in the global simulation time. The main interest in their study is the trend of unitary time versus cell number: when the number of cells overcomes a limit of around 200k cells (this result corresponds to a processor’s cache of 20 MB, being the effect proportional to the cache size), time per cell begins to grow.

Figure 4D also shows the evolution of the parallel fraction in global results generation. The parallel fraction is the time of the operation that linearly depends on the number of processes, calculated using Amdahl’s law. The parallel fraction was estimated using OpenMP for four, eight and 10 threads parallelizing loops over cells and faces. Both global and quadrature cleaning stages produced similar charts. Parallel fraction grows until the same limit and suddenly decreases. This behaviour can be attributed to the overcoming of the cache memory, making the processor communicate directly with main memory (cache increases the parallel fraction and performance due to the use of spatial and temporal locality).

Figure 4C shows the results in the last four stages, which are executed once per iteration, band, and discrete ordinate (150,000 to 750,000 times). The main contribution to global time comes from in-scattering update and differential equations solving stages but, especially in scattering-less simulations, fluxes operations and quadrature results still consume a relevant fraction of time. Quadrature results generation and operation between stages have a similar trend, with a slowly decreasing unitary time. In both stages, there are operations with a linear dependence on the number of cells, and stages with a fixed time, leading to a trend intermediate between the horizontal line of the mesh generation, and a perfect hyperbola of a fixed time stage.

The differential equation solving stage showed the same trend as stages five to seven in Figure 4D but overcame the cache at around 35,000 cells. The in-scattering term update stage shows a new shape with time growing linear from 8,000 to 32,000, and then slowly decreasing. As shown below, its parallel fraction quickly grows until 32,000 cells, having a much slower growth afterwards. This behaviour is caused by the size of different memory cache levels. When the number of cells is small enough, all the data can be stored in cache L1 (a small and fast memory close to the processor, intended to avoid unnecesary repeated queries of data from the main memory), which is the fastest memory. Cache L2 has half the speed of L1 but also uses bigger data burst when reading or writing, which can explain the fast growth of the parallel fraction. Finally, when the operation needs to access the main memory, it behaves like any other operation, with a slow decrease in unitary time and a slow increase in parallel fraction.

## Model optimization and parallelization

### Reduction in memory usage

The first stage in model optimization was the reduction in memory usage. The core of OpenFOAM introduces the class *GeometricField* as a special container storing a value per cell. In this model, only volumetric scalar fields (the template specialization *volScalarField*) are used, meaning 32 or 64 bits (single or double precision) per cell in the domain. As DOM introduces two new discretizations (in wavelength and direction), fields like radiant intensity, stored per cell, discrete ordinate and wavelength band, requires a memory allocation proportional to the product of the three discretization levels. I.e., in a simulation with a 1 M cells mesh, a 15×15 directional discretization (1800 discrete ordinates) and four wavelength bands, solved in double precision, the radiant intensity field would require 53.6 GB (64 bits × 1 M cells × 1800 discrete ordinates × four bands). If the radiation model stores a field once per wavelength band but avoiding the directional discretization, it would require only 244.1 MB (64 bits × 1 M cells × four bands).

Then, the main target in this stage is to minimize the number of fields using the triple discretization. This is especially important in simulations involving radiation transport. As a reference, a fluid dynamics simulation using a k-epsilon turbulence model in the same mesh would require only 53.4 MB of memory allocation (1 M cells, seven volumetric scalar fields). Moreover, reducing memory requirements also improves the simulation speed, as it reduces the number of reading and writing operations. 

The first contribution to memory reduction comes from the storage of the boundary conditions related to the radiation emission sources. The original model uses a volumetric field to store the values of the radiation source term, requiring a scalar per cell and discrete ordinate. This method allocates a huge amount of unnecessary information, such as the values on the internal cells, the values for outgoing ordinates in boundary sources, and repeating values in all directions for isotropic volumetric sources. The developed model replaces this volumetric field by custom storage classes depending on the source type. Isotropic emission sources use a single scalar per boundary, whereas parallel and cone-shaped emission sources use boolean-storage, and Lambertian emission sources use theta-storage. In boolean storage, a single scalar is stored per boundary, and a Boolean (1 bit) per direction (discrete ordinate in the quadrature) and face in the boundary. Theta-storage stores a scalar per theta value ( *θ*) in the quadrature, as the rotation of the quadrature to the central direction of the emission, provides symmetry on the phi angle ( *φ*). All the storage classes developed are based on the definition of sources from user inputs. The user introduces the value of the radiation flux per boundary surface, and the distribution of the radiation emission source terms are calculated to get an average emission flux in the boundary equal to the one provided by the user.

**Figure 5.**
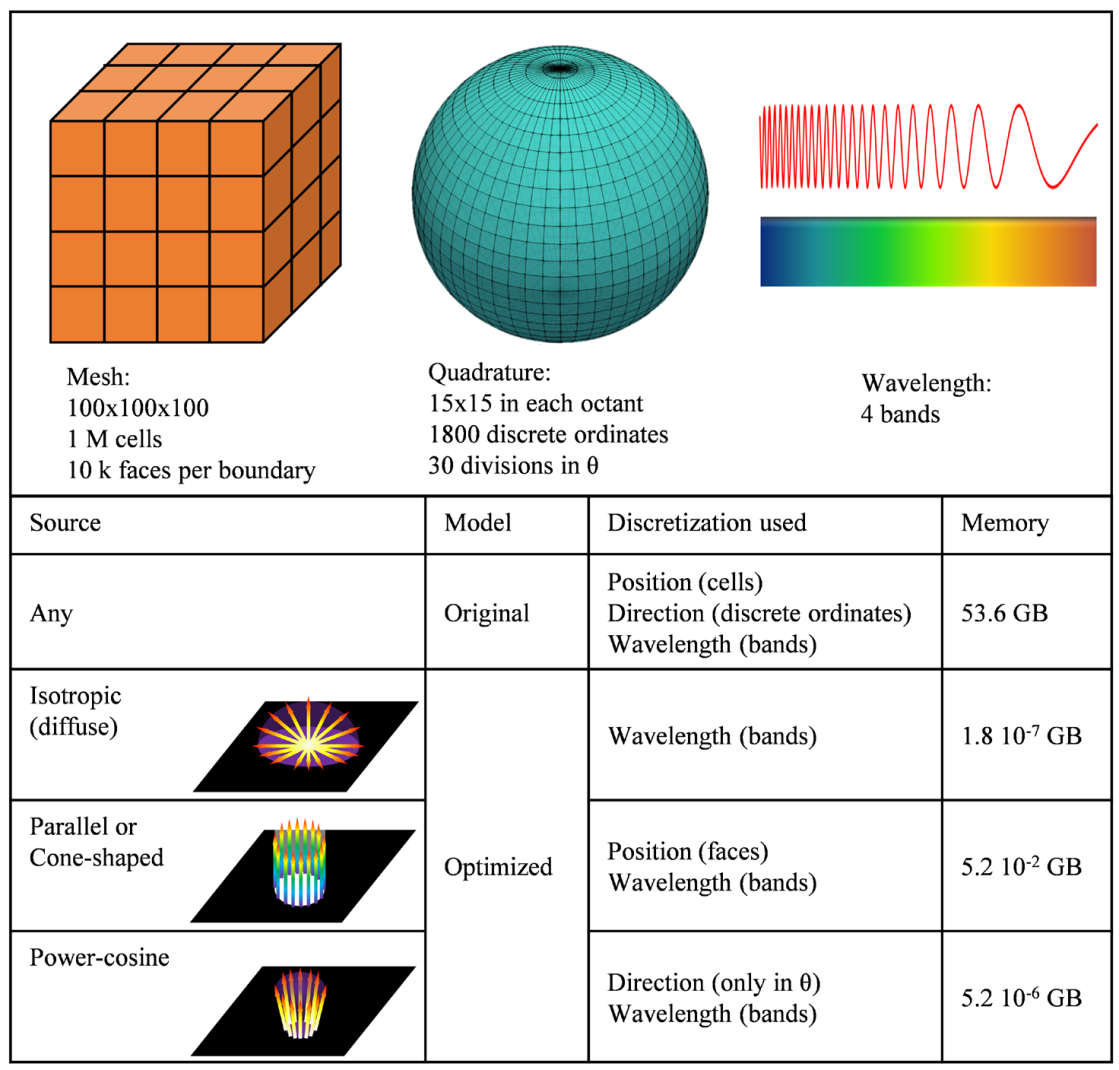
Example of memory requirements in the optimized model as a function of the emission source.

As stated before, reducing the level of discretization used by a field has a significant impact on the memory requirement. Figure 5 shows an example of the reduction in memory allocation for the same simulation when optimizing the model to exploit the specific characteristics of radiation sources, especially for isotropic (diffuse) or power-cosine sources.

The second relevant improvement in the use of memory deals with the storage of the fluxes. The original model stored three different fluxes for each discrete ordinate: emitted flux, incoming flux, and net flux. As the three of them are related (net flux equals incoming flux minus emitted flux), one of them can be omitted. Considering that the net flux is the most relevant result of the simulation, and the incoming flux is necessary for both diffuse reflection and transmission, the emitted flux was the one omitted in the optimized model. Removing the storage of the emitted flux reduces the memory required by fluxes approximately 33% (15% of total memory). On the other hand, the relocation of fluxes from the discrete ordinate class to the quadrature one reduces the memory usage of fluxes a 99.7%, leading to a combined reduction of 99.8%, as shown in Table 2.

**Table 2.** Memory distribution change in main model classes in a 15×15 simulation.

Data to store	Original 8 k cells (Mb)	Optimized 8 k cells (Mb)	Reduction 8 k cells	Original 1 M cells (Gb)	Optimized 1 M cells (Gb)	Reduction 1 M cells
Mesh geometry	1.53	1.53	0.0%	0.19	0.19	0.0%
Radiation sources	142.82	0.51	99.6%	14.56	0.01	99.9%
DO intensity	142.82	142.82	0.0%	14.56	14.56	0.0%
Fluxes	428.47	0.79	99.8%	43.68	0.08	99.8%
Other data	0.48	25.35	-5225.6%	0.05	0.09	-83.1%
**Total**	**716.11**	**171.01**	**76.1%**	**73.05**	**14.94**	**79.6%**

**Figure 6.**
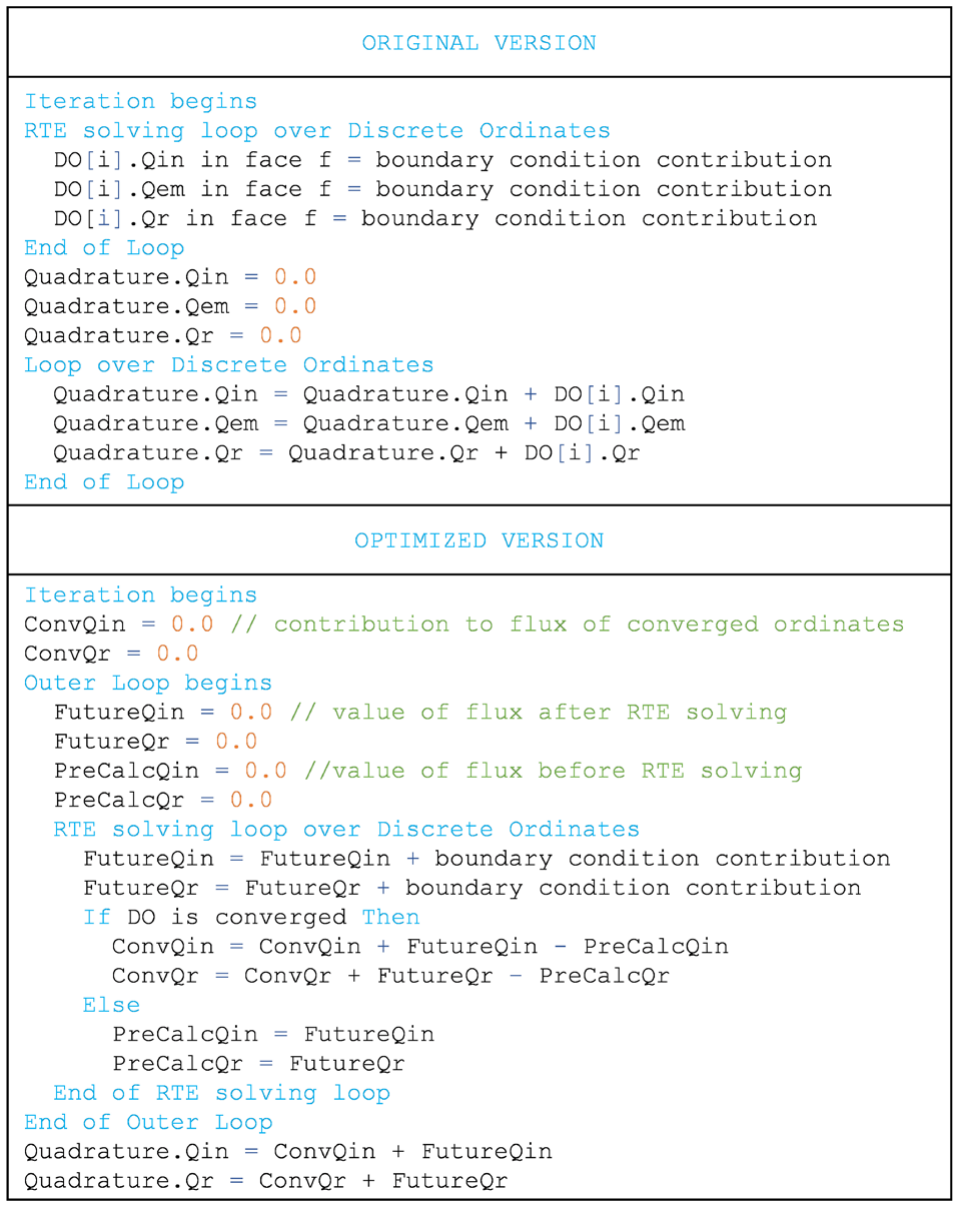
Pseudocode of changes in fluxes update to avoid storage in each discrete ordinate.

**Figure 7.**
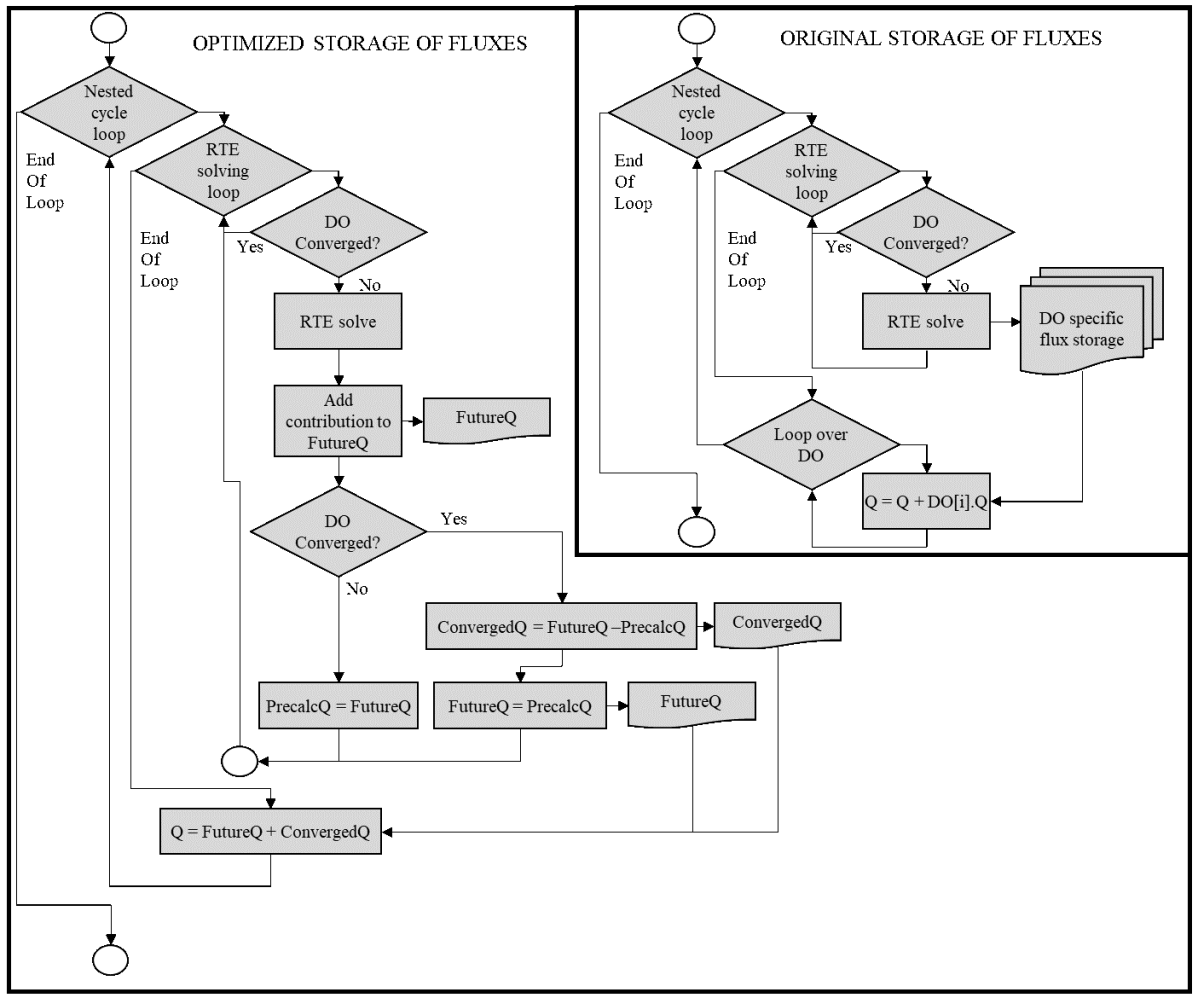
Flux diagram of operations for the storage of fluxes.

The net and incoming fluxes were originally stored in every discrete ordinate and have been transformed to a global value (a single instance compared to 1800 in a reference 15×15 discretization). However, this change hinders the use of nested cycles during resolution. In nested cycles of the resolution, all the discrete ordinates are solved at the beginning of each iteration, and only some of them (those not converged) repeat the solving process without updating the incoming scattering term. In this way, the total number of solving trials per discrete ordinate increases, but the global simulation time is significantly reduced, as it will be shown below. Some operations of results generation are also executed only once at the end of the nested cycles. During nested cycles, each discrete ordinate must update its contribution to the global flux, a simple task in the original model in which the fluxes are stored in each discrete ordinate. This problem is solved in the optimized model by creating a total of four flux fields: *Precalculated*, *Future*, *Converged* and *Total*. After each ordinate solving step, its contribution is stored in Future Flux and then added to Converged Flux (both incoming and net) if it reaches convergence, or to *Precalculated* if not. In the next nested cycle, *Precalculated* flux is restored to zero, while Converged keeps its value. Therefore, Total incoming or net flux can be updated at the end of each nested cycle as the sum of Converged and Precalculated. More detailed pseudocode and flow diagram are shown in Figure 6 and Figure 7.

Table 2 summarizes the effects of memory storage reduction in a 15×15 simulation in 8 k and 1 M cells cases, to observe the trend of memory reduction with the size of the mesh. Combining flux and source storage, the total memory usage reduction is around 75% in a 15×15 standard case. The increase in memory storage in the “Other data” category comes from the anisotropic scattering, included in the model. The relative contribution of each ordinate to each other during scattering is calculated once at the beginning of the simulation, and stored in a NxN matrix, where N is the number of discrete ordinates (1800 in a reference 15×15 simulation). The size of this matrix depends on the number of discrete ordinates, but not on the number of cells, with a minor contribution in high cells number cases, as can be observed in the 1 M cells case.

### Optimization of simulation time

The main stages in the model were optimized and parallelized. The parallelization architecture is a hierarchical combination of the standard DM parallelization (splitting the mesh and solving each piece as a process) and a SM parallelization using OpenMP threads. Threads share the cache memory of processors, dozens of times faster than the main system memory. This memory reads bursts of data instead of the single datum required by the processor. The entire burst can be accessed by threads if they are all working in adjacent data so improving aligned data computations, like arrays, or in this application, a field. As threads work with shared memory, they do not need to communicate with each other to collaborate in concurrent calculations. A total of eight threads were used to profile the reduction of time through parallelization in every example. When hierarchical parallelization is used, the nomenclature M:N means M Distributed Memory computing nodes, each of N Shared Memory processing threads. When individual operations are analysed, SM parallelization is used. 

The first optimized stage was the generation of quadrature results. Operations with fluxes were parallelized and limited only to boundaries, as their value is always zero in every internal cell. Incident radiation update operation was parallelized and it keeps running only once per iteration after nested cycles of resolution.

**Figure 8.**
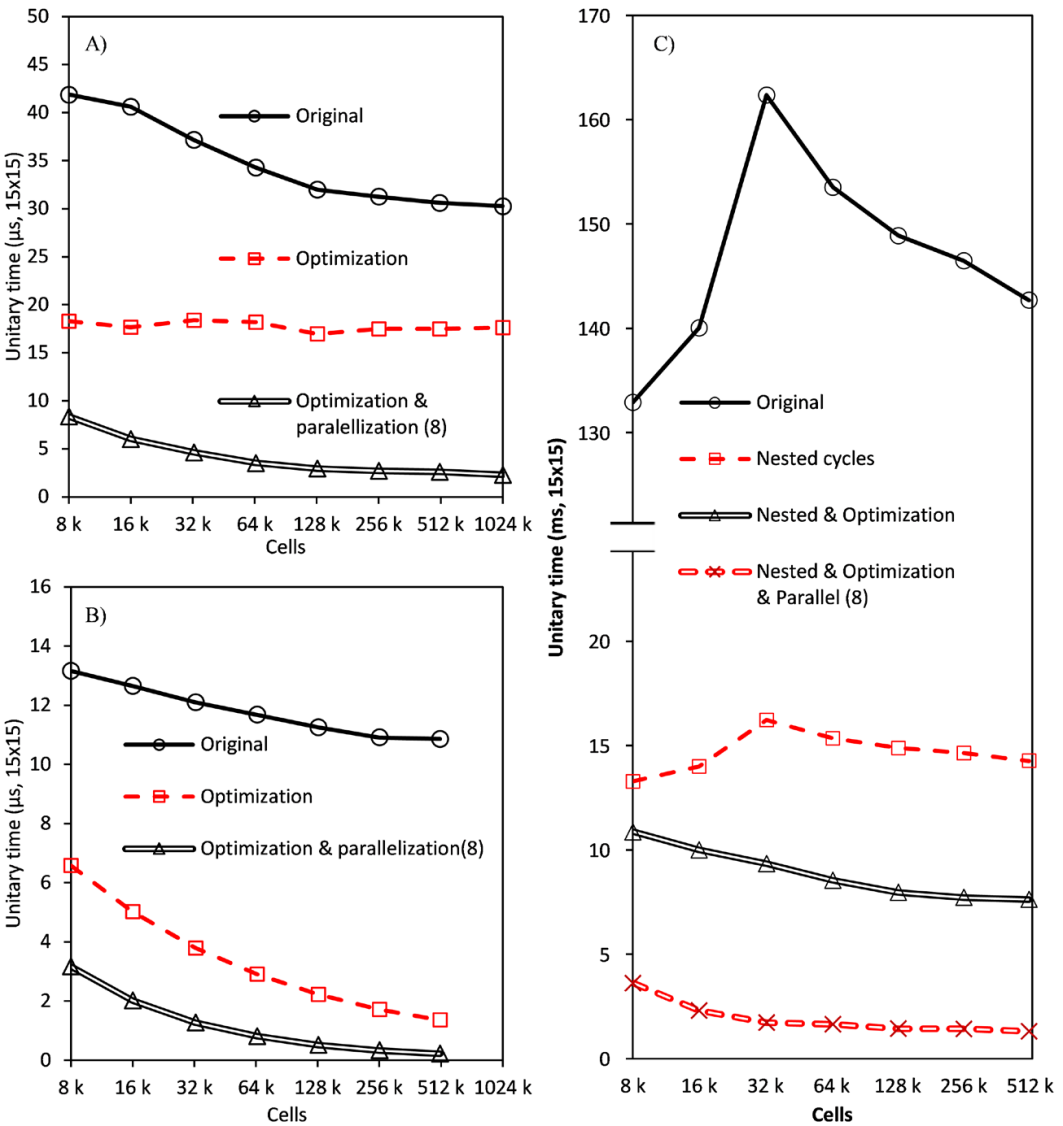
Performance enhancement in every algorithm stage: Quadrature results generation ( **A**), operations between fluxes ( **B**) and incoming scattering term update ( **C**).

Figure 8A shows the effect of optimization in the quadrature results generation stage on the unitary time, calculated dividing the operation time by the number of cells in the mesh. The results confirm an average speedup (ratio between the computational time of the original and enhanced models) of 1.95 when memory-optimized, 5.11 if also parallelized.

Figure 8B shows the results corresponding to the operations with fluxes, with a speedup of 4.44 and 19.7, for the optimized and optimized and parallelized models, respectively.

During the calculation of the incoming scattering term for each discrete ordinate in each cell, the contribution of all the rest of the ordinates must be calculated. This is a triple loop where, in order to gain the best improvement of a memory aligned computation, the inner level (loop over cells) was parallelized. The operation has also been limited to cells, avoiding unnecessary calculus in boundaries, and the storage and access to scattering matrix have been optimized using pointers arithmetic over a single array instead of original nested arrays.

A reference to the beginning of the array is created and forwarded after completion of the inner loop to the next row of the matrix, in the same array. This avoids operations to calculate the index, which is a trivial task, but performed millions of times. This change increases the speedup generated by the change in storage from 1.53 to 1.684 (a 10% increase). This improvement disappears if the intermediate or outer loops are parallelized instead of the inner one.

Finally, it is performed only once per iteration, before nested cycles begin. Figure 8C shows the effect of nested cycles (speedup of 10), the addition of optimization (speedup of 16.84), and finally, the total reduction when parallelization is included (speedup of 85.53).

**Figure 9.**
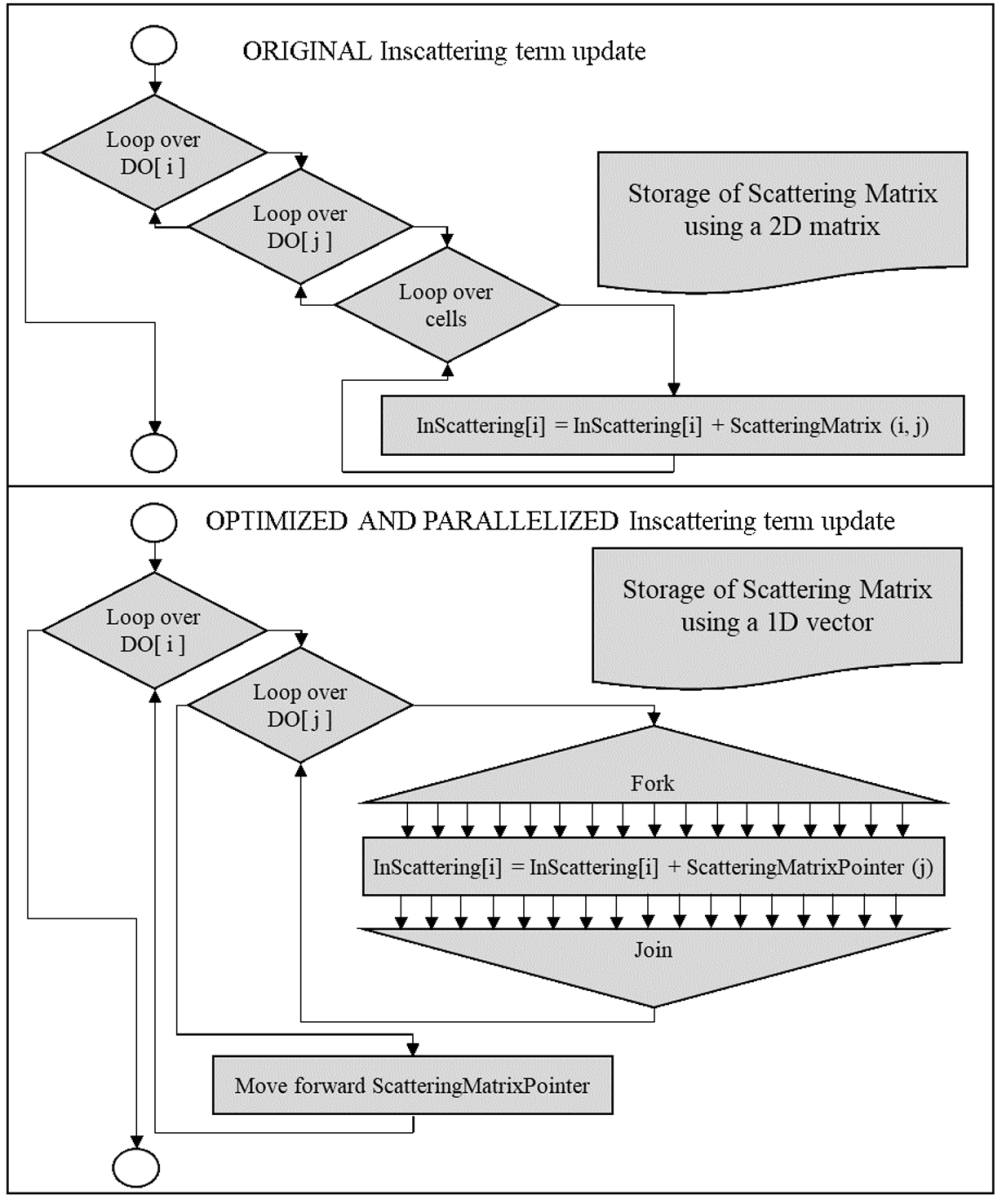
Flux diagram of the in-scattering term update stage.

Table 3 summarizes the effects of optimization and parallelization on the computational time per iteration. A more detailed flow diagram can be found in Figure 9. As it can be noticed, despite the significant increase in the computational resources required for the differential equation solving stage in each iteration (it is solved once per sub-iteration in nested cycles), the global time of the simulation is significantly reduced. Further improvement in the differential equation solving stage would require the parallelization of the OpenFOAM’s core functions, involved not only on the radiation transport calculations but also in any other module such as fluid-dynamics, mass transfer or chemical reaction.

**Table 3.** Effect of optimization and parallelization in the last four stages in the 256 k cells mesh.

	Original		Optimized		Optimized & parallelized (8)	
Stage	Time (s)	Contribution	Time (s)	Contribution	Time (s)	Contribution
Incoming scattering update	142.697	95.21%	84.737	54.24%	16.684	18.93%
Radiative transfer eq. solve	7.144	4.77%	71.445	45.73%	71.445	81.06%
Quadrature results generation	30	0.02%	16	0.01%	6	0.01%
Operations between fluxes	11	0.01%	24	0.02%	6	0.01%
Global iteration	191		196		100	
Equivalent iteration	191		39.2		20	

RTE, radiative transfer equation.

Optimized version slightly increases the time per iteration but, as shown in the application of the model below, nested cycles significantly reduce the number of iterations needed to reach the same convergence criterium (~80% reduction). This means that every optimized iteration corresponds to approximately five original iterations. Therefore, the “equivalent iteration” time would be of 39.2 s when optimized (a global speedup of 4.87), and 20 s when optimized and parallelized (speedup of 9.55).

### Model scalability

One of the expected outcomes of the optimized model is the increase in the scalability, meaning that a higher amount of computational resources can be applied to the same simulation, with a growing performance. The limit in scalability in standard parallelization with the optimized OpenFOAM model was compared with that achieved using ANSYS Fluent in their respective optimal setup under the same configuration in terms of mesh, schemes and parallelization scheme (X DM : 1 SM), except for the communication architecture between nodes. ANSYS Fluent uses a master-slave architecture while OpenFOAM uses a peer-to-peer one. This difference is not a result of the present work, but sets a different starting point for the improvement of scalability produced by the DM:SM hierarchical parallelization. Both software programs (OpenFOAM v6 and ANSYS Fluent v2019 R2) have been run on equivalent workstations comprised of a dual Intel Xeon E5-2630 processor and 64 GB RAM.

To study this limit, the three case studies presented below (real photoreacting systems with complex geometry and different mesh densities) were simulated to sample the reduction in time as the number of processes grow. Simulation time changes from one case to another, but the speedup (ratio between original time and parallelized time) suffers minor diversions and thus, the average speedup value is presented. This study neglects the increase in time produced by a slower convergence when the number of mesh divisions grows.

**Figure 10.**
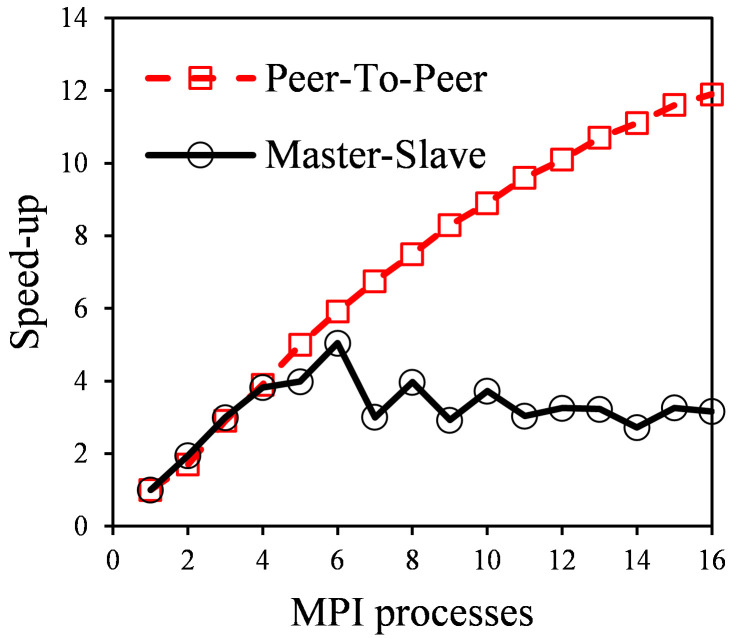
Simulation time trend in Master-Slave communication (ANSYS Fluent) and Peer-To-Peer communication (OpenFOAM).

The results ( Figure 10) show significantly different trends between both software. While ANSYS Fluent reached the expected maximum peak in six nodes with a lower performance afterwards, OpenFOAM speed kept growing until 16 nodes. This is caused by different communication architecture during DM parallelization. ANSYS Fluent uses a “Master-Slave” architecture, where all the messages are between a single node (the master) and one of the remaining nodes (slaves). Then, each node sends its interface values to the master (except for the master itself) and receives the interface values of its neighbour. OpenFOAM uses a “Peer-To-Peer” communication architecture, where every node communicates directly with its neighbours.

As an example, if a cylindrical system is divided into ten slices along its central axis, with each node having two interfaces except the endings having one, the number of messages sent by the master in “master-slave” will be 18, while in “peer-to-peer” endings will send a single message and central nodes two each, as they communicate only to neighbour processes. The maximum communication time in a node grows linear in “master-slave” and is almost constant in “peer-to-peer”.

## Applications of the optimized model

The performance of the optimized model has been evaluated in different scenarios using ANSYS Fluent CFD software as a benchmark. All the simulations have been developed using the same numerical schemes and model configurations in both software programs. To get a high precision in the resolution of the RTE, the chosen configuration was:

- 15×15 angular discretization, required to capture adequately specular reflection and direct sunlight.
- A Green-Gauss cell-based linear upwind scheme, required for high absorption media.
- Convergence with a residual target value of 10 ^-6^.

Depending on the system, some simulations could be significantly faster using simpler schemes, but the same configuration was chosen in all the reactors and software for comparison purposes. In any case, as the implementation of the numerical methods is similar in both software, acceleration of the simulation with other model configuration would be equivalent, leading to the same conclusions in relative terms.

### Solar water disinfection

**Figure 11.**
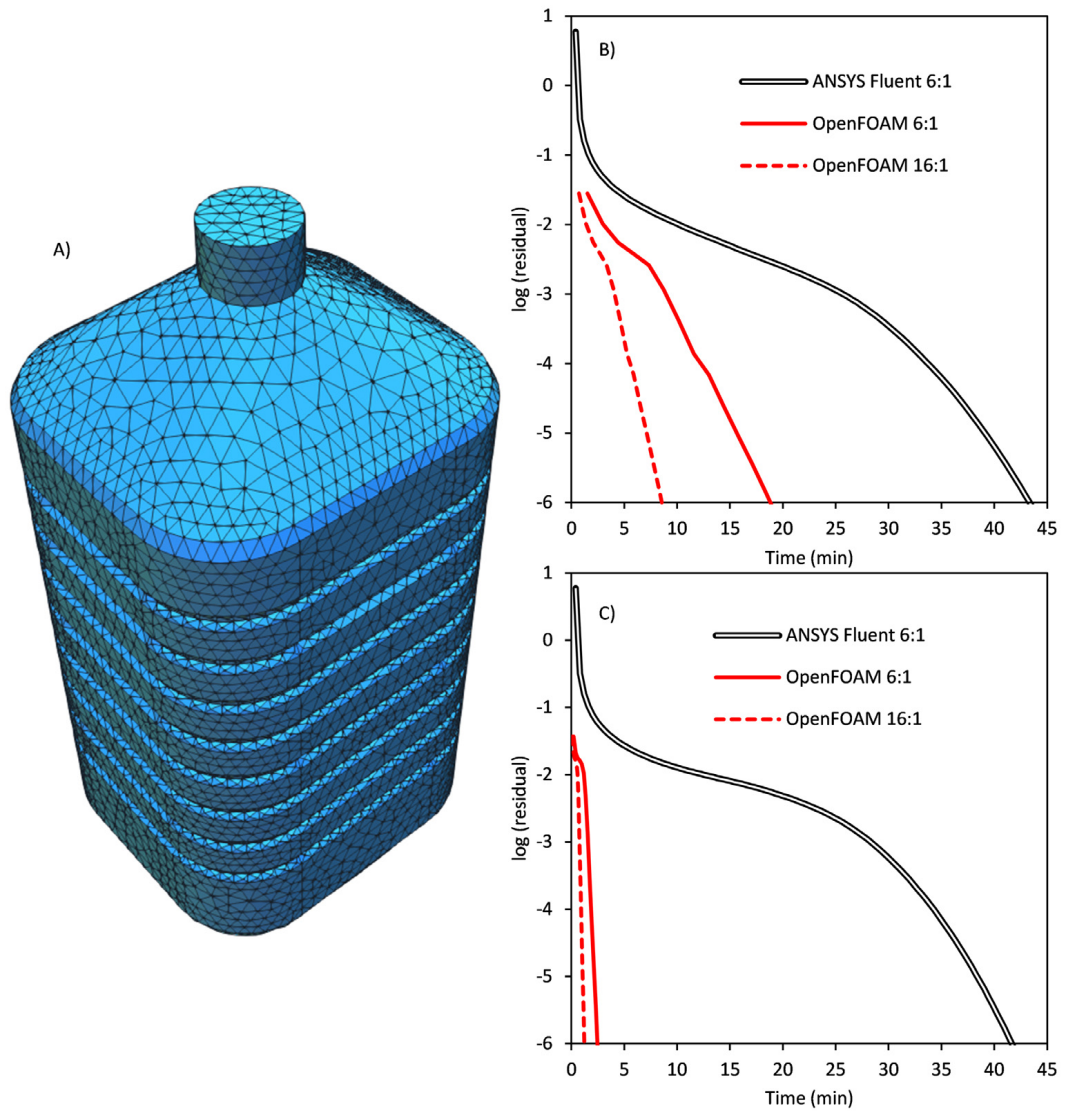
**A**) Geometry and meshing of the high-capacity solar disinfection container. **B**) Evolution of the residuals in the simulation with isotropic diffuse solar light illumination. **C**) Evolution of the residuals in the simulation with parallel direct solar light illumination.

Solar water disinfection processes are based on the inactivation of microbial pathogens present in water by the UV photons of solar light. When this process is carried out in high-capacity containers, the estimation of the radiation distribution becomes critical to predict the required exposure time ^20^. Figure 11A shows the meshed geometry of the simulated high-capacity container.

This setup, with a low number of cells and no scattering, corresponds to the least favourable situation to analyze the improvement in the model performance. Therefore, it represents the minimal improvement as a reference.

Solar disinfection of clear water is the simplest scenario, as it can be assumed that water is a non-participating media (no absorption, no scattering). Therefore, most of the significant improvements in the optimized model (focused on scattering) don’t apply, and thus the parallel configurations only use distributed computation in six DM and 16 DM. Figure 11B and 11C show the results of the simulation with an incident radiative flux of 100 W m ^-2^ under two different illumination conditions of diffuse solar light and direct solar light. In both cases, the evolution of the residuals until reaching convergence is monitored in three computational setups: ANSYS Fluent fastest parallelization (six DM nodes), the same configuration in OpenFOAM and the fastest configuration in OpenFOAM (16 DM single-threaded nodes). For diffuse solar light ( Figure 11b), with six parallel processes, ANSYS Fluent required a total of 100 iterations in 43.72 min while the OpenFOAM optimized model needed 14 iterations and 19.67 min, a speedup of 2.23. The apparent reduction in iterations is, in fact, an increase, as due to nested cycles every iteration in OpenFOAM is solving ten times the RTE in each ordinate (a total of around 140 iterations). The simulation with 16 DM processes in OpenFOAM needed 15 iterations and 8.9 min to converge. Each process solves RTE in its region using implicit values, while interface values are used explicitly, slowing the convergence as they grow. This explains the extra iteration and introduces an additional limit to DM parallelization performance.

For direct parallel radiation ( Figure 11c), the absence of scattering and reflection, leads to a single discrete ordinate carrying radiation, the one of the incident light. While the standard DOM in ANSYS Fluent keeps solving every direction in every iteration (a total computational time of 41.75 min and 100 iterations using six parallel processes), in the optimized OpenFOAM model converged ordinates are solved only once at the beginning of each iteration, while not converged ordinates are solved ten times. This led to a total of 13 iterations in OpenFOAM six DM processes, with a total computation time of 2.48 min, a speedup of 16.8. Again, pure DM parallelization resulted in the fastest configuration in OpenFOAM, reaching convergence in 13 iterations and a total computational time of 1.22 min for 16 parallel processes, a speedup of 34.2. This significant reduction in the simulation time has not only quantitative effects, but also qualitative consequences, opening the possibility of real-time transient simulations of solar driven processes using a high precision DOM.

### Photocatalytic processes with tubular lamps

**Figure 12.**
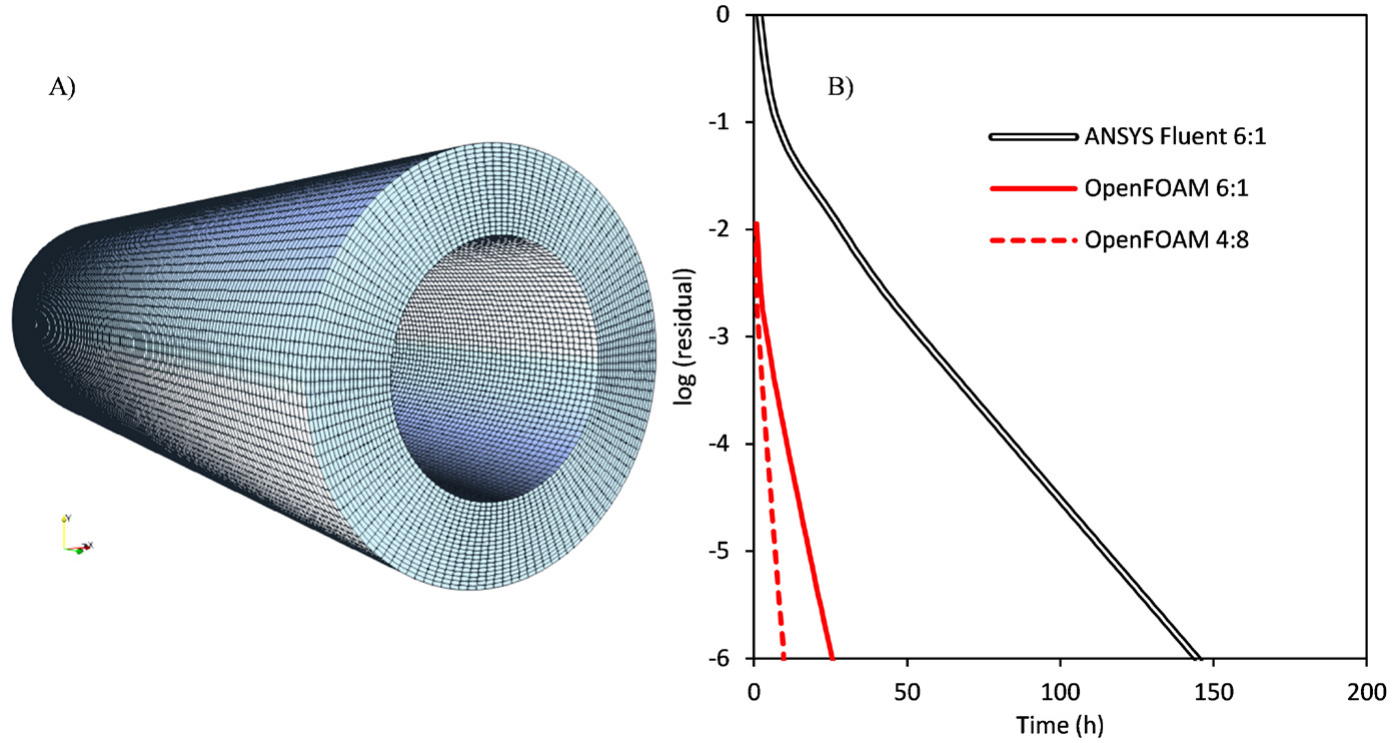
**A**) Geometry and meshing of the annular photocatalytic reactor. **B**) Evolution of the residuals in the simulation of the radiation field with a catalyst loading of 0.1 g/L of P25 TiO _2_.

Photocatalytic processes are advanced oxidation technologies able to remove refractory chemical contaminants and microbial pathogens in water and air by means of the photoactivation of a heterogeneous semiconductor acting as catalytic material ^21^. TiO _2_ is by far the most common photocatalytic material, requiring UV-A illumination for the activation ^22^. Mercury lamps have been widely used for years as a source of UV light in photoactivated processes ^23^, and due to their tubular nature, annular reactors are the optimal configuration to improve the use of the light. Figure 12A shows the geometry of the annular photocatalytic reactor and its structured mesh composed of 368,440 hexahedral cells used for the simulation. Assuming a perfect mixing and dispersion of the photocatalyst particles, the medium can be considered pseudo-homogeneous, with constant volumetric absorption and scattering coefficients that can be calculated for a catalyst loading of 0.1 g/L from the specific coefficients reported in the literature. The anisotropic scattering phase function (Henyey-Greenstein) was also taken from the literature ^24^. Every boundary was declared as transparent, setting an isotropic diffuse radiation emission source in the inner face of the annulus with a radiative flux of 100 W m ^-2^.

Figure 12B shows the evolution of the residuals trend along simulation time. In comparison with the solar disinfection processes, the presence of anisotropic scattering leads to significantly higher computational time, also increased by the finer mesh, with approximately three times more cells, required to capture the radiation profiles in the annular volume. The results show that ANSYS Fluent simulation in its optimal configuration of six DM processes required 104 iterations to reach convergence, and 145.25 h (aprox. six days). The optimized OpenFOAM model needed 28 iterations and 26.35 h (speedup of 5.51) in the same configuration of six DM processes of a single SM thread. 16 DM processes (included as *Underlying data* ^9^) led to a speedup of 11. In comparison, the optimal configuration for this simulation was four DM processes of eight SM threads each, with 27 iterations and 9.81 hours (a total speedup of 14.8). The change in the optimal configuration is caused by the presence of light scattering. In a single DM process, time keeps decreasing until 16 SM threads (the total number of threads present in each processor of the server), but the simulation in two DM processes of 16 SM threads each was slower than the 16 DM processes case. Hardware limitations hindered a configuration of more than 32 total threads, but DM parallelization is not affected by multithreading, with effective scalability more than ten times higher.

### Photocatalytic processes with solar collectors

**Figure 13.**
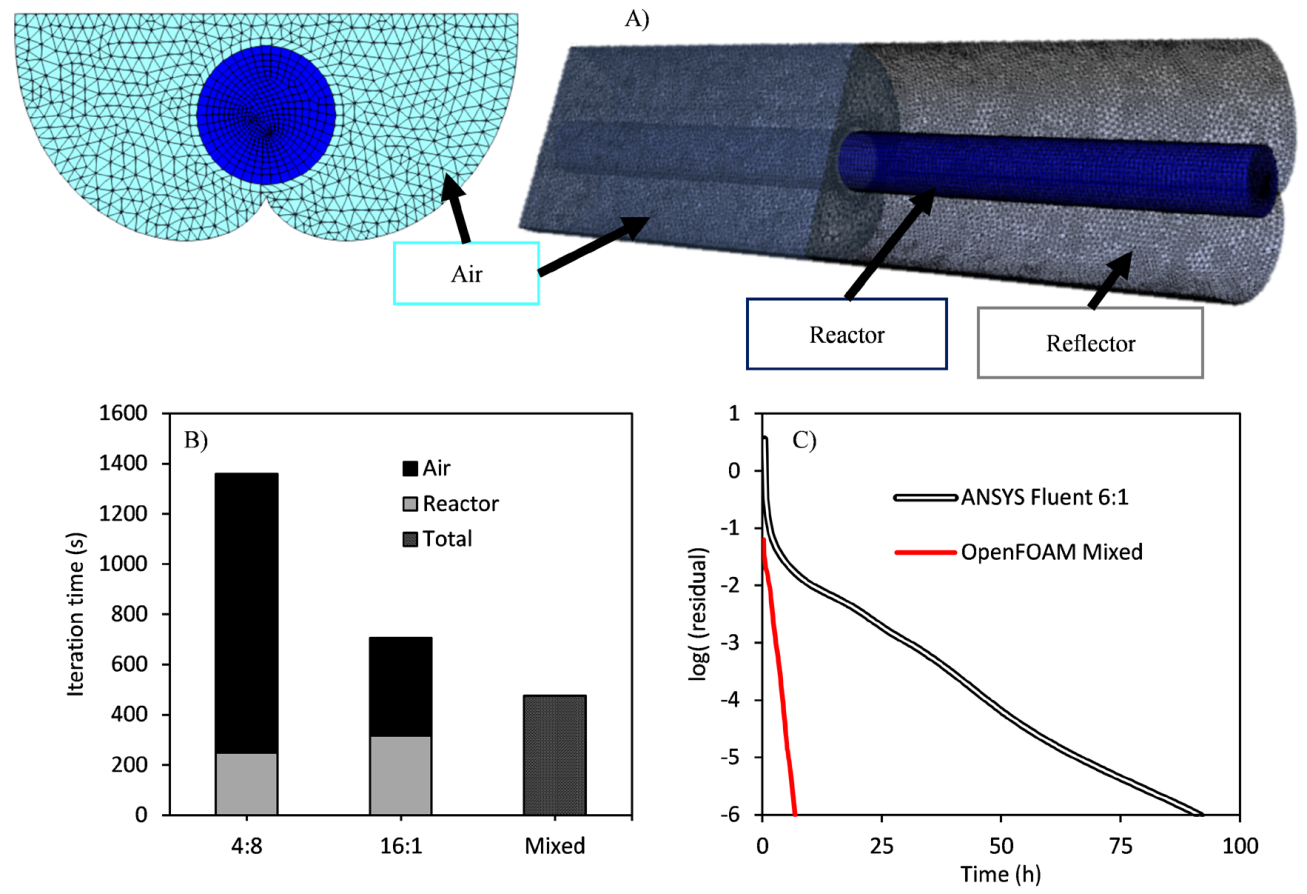
**A**) Geometry and meshing of the tubular photocatalytic reactor coupled to a compound parabolic collector (CPC) solar collector. **B**) Computational time for the resolution of the two different regions as a function of the parallelization scheme. **C**) Evolution of the residuals in the simulation of the radiation field with a catalyst loading of 0.1 g/L of P25 TiO _2_.

The last scenario used for the evaluation of the optimized model consists of the simulation of photocatalytic processes using solar collectors. In these systems, together with the high absorption and scattering in the tubular reactor, reflection phenomena in the collector surface and transmission in the transparent air region between the collector and the reactor have to be considered. Compound parabolic collectors (CPCs), are commonly used for solar water treatment applications ^25^ ( Figure 13A). These systems are designed to focus on the tube both direct and diffuse sunlight, and, to get the average optical operation along the year, they are operated in a static position faced to the equator with a tilted angle equivalent to the local latitude.

Figure 13A shows the geometry and mesh of the studied solar compound parabolic collector coupled with a tubular photoreactor, with two well-defined independent regions of ~80,000 cells in the tube and ~600,000 cells in the air region. The simulation of these two coupled regions (they cannot be solved alone), having entirely opposite optical properties, makes the choice of the optimal parallelization setup more challenging. The transparent air region would benefit from multiple processes of a single thread, while the reactor region would show a higher performance in a reduced number of processes of many computing threads. Although the reactor region has less than 15% of total cells, the average resolution time of both regions is comparable, due to the increase in time associated with the scattering in the reactor.

Three different configurations were tested in this simulation:

a) Four DM processes of eight SM threads each, ideal for the tube reactor (scattering media),

b) 16 DM processes of one SM thread each, ideal for the air region (transparent media),

c) A mixed configuration, using two DM processes of eight SM threads each for the reactor region, and eight DM processes of a single SM thread for the air region.

The results of the computational time required for the resolution of the two different regions as a function of the parallelization scheme are shown in Figure 13B. Four DM processes of eight SM threads each led to the lower computational time for the reactor region, but it shows a minor effect in the air region, and therefore the global simulation time is significantly higher than for the other two configurations. A two-fold decrease is observed when using the optimal configuration for the air region (16 DM processes of a single SM thread each). Finally, a three-fold decrease in the simulation time is obtained when using the mixed configuration, with the optimal parallelization scheme for each region.

In configurations a) and b) both regions are solved in serial mode, using the same configuration. In the example, in configuration b), the air region is divided into 16 pieces, each solved by a process, and the same process solves then its piece of the reactor region. In contrast, for configuration c) both regions are solved at once, using different processes. Pieces are bigger, but there are also fewer interfaces, reducing the communication time, and the iteration needed for convergence. This configuration clearly led to the best performance.

Finally, Figure 13C shows the comparison of this optimal mixed configuration of the parallelized OpenFOAM with the standard DOM results with the optimal ANSYS Fluent configuration of six processes of a single thread. The speedup in this reactor was 12.73. Configuration a) (4:8), and configuration b) (16:1) led to speedup values of 4.63 and 8.37, respectively.

## Conclusions

The optimization and parallelization of the DOM model in OpenFOAM reduced dramatically the required computational cost of radiation transport simulation in complex systems such as photoactivated processes. The reduction in memory use of around 75% allows a more rigorous discretization in any direction and/or space. Every iteration time was reduced by 40%, while the total number of iterations decreased by 80%.

Shared memory parallelization shows higher efficiency than distributed memory approaches, and its main drawback, the lack of scalability, was avoided using a mixed hierarchical combination. The comparison between the optimized model and the standard DOM available in ANSYS Fluent in three different applications has shown significant improvements in the simulation speed, ranging from five- to 35-fold decreases in the simulation time. These results also point to the significant advantage derived from the selection of the optimal parallelization architecture as a function of the optical properties of the medium.

The developed optimized model represents a huge improvement in the capabilities of radiation transport simulation in OpenFOAM. The increase in the model speed and its scalability makes it suitable to tackle new challenges, such as transient radiation transport simulation, or even real-time simulation. Finally, this work also constitutes a starting point for the global implementation of combined parallelization in all the OpenFOAM models.

## Data availability

### Underlying data

Zenodo: Dataset of Paper “Optimization and parallelization of the Discrete Ordinate Method for radiation transport simulation in OpenFOAM: Hierarchical combination of shared and distributed memory approaches”. https://doi.org/10.5281/zenodo.4434729 ^9^.

This project contains the following underlying data:

– Paper3_01.csv (time profiling of the DOM model stages, dataset details can be found in Paper3_01.odt)
– Paper3_02.csv (speedup and parallel fraction of the “global results generation” stage in the DOM model, dataset details can be found in Paper3_02.odt)
– Paper3_03.csv (comparison of computational time between original and modified DOM model stages, dataset details can be found in Paper3_03.odt)
– Paper3_04.csv (scalability of the peer-to-peer communication (OpenFOAM) and master-slave communication (ANSYS Fluent) architectures, dataset details can be found in Paper3_04.odt)
– Paper3_05.csv (results of the benchmarking of the model with ANSYS Fluent in three reactors, dataset details can be found in Paper3_05.odt)
– Paper3_06.zip (mesh of the jerrycan, dataset details can be found in Paper3_06.odt)
– Paper3_07.zip (mesh of the annular reactor, dataset details can be found in Paper3_07.odt)
– Paper3_08.zip (mesh of the tubular reactor couple to a compound parabolic collector, dataset details can be found in Paper3_08.odt)

Data are available under the terms of the Creative Commons Attribution 4.0 International license (CC-BY 4.0).

## Software availability

Source code available from: https://github.com/PhotonersURJC/OpenFOAM_DOM_Parallel/tree/DOMv2.0

Archived source code at time of publication: https://doi.org/10.5281/zenodo.4296511 ^26^.

License: GNU General Public License v3.0
