# Supplementary material for: Optimization and parallelization of the discrete ordinate method for radiation transport simulation in OpenFOAM: Hierarchical combination of shared and distributed memory approaches
Source: Open Res Eur. 2021 Mar 24;1:2. [Version 1] doi: 10.12688/openreseurope.13017.1 (PMC10445830; doi:10.12688/openreseurope.13017.1)
Supplement: Peer review history

# Peer review: Reviewer response for version 1

**DOI:** 10.21956/openreseurope.14087.r27042

**Published:** 2021-06-15

**Authors:** Eugene de Villiers, George Karpouzas

**Affiliations:** 1 Engys Hellas, Athens, Greece; 2 Engys Ltd, London, UK

In this manuscript, several code optimization are presented to improve the existing Discrete Ordinate Method (DOM) inside the OpenFOAM framework  for simulating radiation transport problems.

 As the paper clearly describes, these improvements are accomplished using state-of-the art coding techniques commonly used in the field of computer science. These consist mainly of: 1) refactoring data structures to reduce memory requirements and cache misshits and 2) by activating multithreading techniques which leads to improved performance. The improvements are added to the solution process in a step-by-step way, which helps the reader to understand the contribution and importance of every element.

 The optimized DOM code is compared with the original (OpenFOAM) and a commercial software (Ansys Fluent) and the performance gains reported are quite impressive. The cases and data from these comparisons are publicly available to reproduce.

 Based on the above, the manuscript should be accepted for indexing.

Is the rationale for developing the new method (or application) clearly explained?

Yes

Is the description of the method technically sound?

Yes

Are the conclusions about the method and its performance adequately supported by the findings presented in the article?

Yes

If any results are presented, are all the source data underlying the results available to ensure full reproducibility?

Yes

Are sufficient details provided to allow replication of the method development and its use by others?

Yes

Reviewer Expertise:

CFD and Mesh generation code development.

We confirm that we have read this submission and believe that we have an appropriate level of expertise to confirm that it is of an acceptable scientific standard.

# Peer review: Reviewer response for version 1

**DOI:** 10.21956/openreseurope.14087.r26529

**Published:** 2021-05-10

**Authors:** João Miguel Nóbrega

**Affiliations:** 1 Institute for Polymers and Composites/i3N, University of Minho, Guimarães, Portugal

The submitted manuscript presents a relevant piece of work performed to improve the performance of an open-source solver developed to solve the Radiative Transfer Equation, following the Discrete Ordinate Method. The results obtained show clear improvements not only in terms of memory requirements but also in calculation time.

 All the developments performed are clear and described in detail. Moreover, the results obtained in the provided case studies, which are compared with similar ones done with proprietary software, clearly evidence the advantages of the novel approaches.

 All the codes developed and case studies are publicly accessible, which allow the reproducibility of all the information provided.

 Based on the above, the manuscript should be accepted for indexing in its present form.

 For the final version the authors are just advised to improve the graphical resolution of some of the figures, namely: Figures 7, 9 13A).

Is the rationale for developing the new method (or application) clearly explained?

Yes

Is the description of the method technically sound?

Yes

Are the conclusions about the method and its performance adequately supported by the findings presented in the article?

Yes

If any results are presented, are all the source data underlying the results available to ensure full reproducibility?

Yes

Are sufficient details provided to allow replication of the method development and its use by others?

Yes

Reviewer Expertise:

Computational Modelling.

I confirm that I have read this submission and believe that I have an appropriate level of expertise to confirm that it is of an acceptable scientific standard.
